# Frequent Changes of the Underclothing

**Published:** 1881-05

**Authors:** 


### FREQUENT CHANGES OF THE UNDERCLOTHING.

Weak patients should change their underclothing as seldom as possible, as every change robs the surface of a portion of the oil that is necessary to keep the skin soft and lubricated, and to make it a non-conductor of heat. When the skin is in an oily condition, as is found in the healthy, the liability to be affected by colds is much less than when it is in the rough and dry condition.

The oily state of surface, as is usually found with the healthy individual, is maintained by many thousands of sebaceous glands that are located in the integument; when a patient is in a weak condition from catarrhal disease, these glands do not supply this important non-conductor as abundantly as the skin requires it. For this reason, those patients that are thin in flesh, and on the surface of whose body there is little or no oily material, should not change the stocking-knit suit that is next to the body until it has become soiled, which may be in about one, two, three or more weeks. The weaker the patient, the less frequently should changes take place, and the less frequently will they require to take place, as the dry skin does not soil the clothing so rapidly as does the healthy oily skin.

If the suit next to the body does not cause undue perspiration during the night, it should be worn at this time as well as during the day.

The supplementary suits should not be permanently removed until the weather becomes warm in the spring. The last supplementary suit—leaving the one thin stocking-knit suit next to the body—may usually be removed about the 15th of June.

Patients must bear constantly in mind that it is far preferable to suffer the temporary discomfort that is occasioned by the presence of the extra suits on occasional warm days, that sometimes occur before warm weather has permanently set in, than to risk taking a cold by their too early removal. In other words, it is preferable to bear patiently the inconveniences caused by the heat, than to suffer several days' or maybe weeks' sickness, the result of the too early removal of the underclothing.—*Dr. Rumbold's Hygiene of Catarrh.*

